# Dépistage sérologique de SARS-CoV-2 chez une population de personnel de santé à Nouakchott-Mauritanie

**DOI:** 10.11604/pamj.2021.38.55.24259

**Published:** 2021-01-18

**Authors:** Mohamed Lemine Ould Salem, Mohamed Ahmed Med Mahmoud Sidiya, Ahmed Baba Ahmedou Eibih, Mohamed Mahmoud Maouloud, Brahim Hamad Ngaide, Leila Dedy, Lalla Mariem Hamza, Fatimetou Yacoub

**Affiliations:** 1 Laboratoire de Biologie médicale du Centre Hospitalier National de Nouakchott, Nouakchott, Mauritanie,; 2 Faculté de Médecine de Nouakchott, Nouakchott, Mauritanie

**Keywords:** SARS-CoV-2, COVID-19, amplification en chaîne par polymérase, test de diagnostic rapide, Nouakchott, Mauritanie, SARS-CoV-2, COVID-19, polymerase chain reaction, rapid diagnosis test, Nouakchott, Mauritania

## Abstract

La première flambée de contagion respiratoire due à une étiologie inconnue a été signalée dans la ville chinoise de Wuhan décembre 2019. L´Organisation Mondiale de la Santé (OMS), a utilisé le terme « nouveau coronavirus 2019 » le 29 Décembre 2019. Cette épidémie qui est actuellement appelée Coronavirus 2 du syndrome respiratoire aigu sévère (SARS-CoV-2), la maladie causée par le SARS-CoV-2, a été par la suite appelée par l'OMS: maladie à Coronavirus 2019 ou COVID-19. L´objectif de cette étude était de déterminer la prévalence des anticorps anti SARS-CoV-2 chez tous les employés du centre hospitalier national de Nouakchott (CHN). L´étude a été menée pendant la semaine du 20/05/2020 au 27/05/2020 chez 853 employés tous grades confondus (médecins, pharmaciens, infirmiers, secrétaires, personnels de sécurité, administrateurs…) dont 504 de sexe masculin et 331 de sexe féminin soit un sex- ratio de 1,52 avec un âge moyen de 39 ans et des extrêmes d´âge de 20 ans et 60 ans. Ce dépistage à la recherche des anticorps IgG et IgM anti-SARS-CoV-2 a été réalisé par la technique immuno-chromatographique Biotime (Xiamen Biotime Biotechnology Co., Ltd.). Parmi les 835 employés inclus dans notre étude, 14 étaient positifs (soit 1.67%) dont 12 avaient des anticorps anti-SARS-CoV-2 de type IgM et IgG et deux (2) n´avaient que des IgM isolées. L’amplification en chaîne par polymérase (PCR) sur écouvillonnage naso-pharyngé, a été réalisée chez ces 14 patients et s´est révélée positive chez six. Si la PCR constitue le gold standard pour le diagnostic du SARS-CoV-2, les tests sérologiques à la recherche des anticorps anti SARS-CoV-2 particulièrement les tests de diagnostic rapide (TDR) constituent un complément diagnostic du COVID-19. Ils ont l´avantage de la facilité de réalisation, de leur sécurité aussi bien dans le laboratoire, dans les services cliniques ou en ville. Les TDR nous ont permis de détecter des porteurs du SARS-CoV-2 au sein des employés au CHN, ce qui a permis de les prendre en charge et de les isoler, en fonction des résultats de leurs PCR, pour protéger les patients ainsi que leurs entourages.

## Abstract (en)

The first outbreak of epidemic respiratory disease due to unknown etiology was reported in the Chinese city of Wuhan December 2019. The World Health Organization (WHO) firstly used the term “new coronavirus 2019” on December 29, 2019. This pandemic, which is currently called severe acute respiratory syndrome coronavirus 2 (SARS-CoV-2), is a disease caused by SARS-CoV-2. It was subsequently called coronavirus disease 2019 (COVID-19) by the WHO. The purpose of this study was to determine the prevalence of antibodies against SARS-CoV-2 in all employees of the Nouakchott National Hospital Center (CHN). The study was conducted during the week 20/05/2020 to 27/05/2020. It involved 853 employees of all ranks (doctors, pharmacists, nurses, secretaries, security personnel, administrators...) of whom 504 were male and 331 were female, with a sex ratio of 1,52 with an average age of 39 years, ranging from 20 to 60 years. The screening for IgG and IgM antibodies to SARS-CoV-2 was performed using Biotime (Xiamen Biotime Biotechnology Co., Ltd.) immunochromatographic technique. Out of 835 employees included in our study, 14 were positive (1.67%) of whom 12 had IgM and IgG anti-SARS-CoV-2 antibodies and 2 had isolated IgM. Nasopharyngeal swab polymerase chain reaction (PCR) was performed in these 14 patients and was positive in six. While PCR is the gold standard for the diagnosis of SARS-CoV-2, serological tests for SARS-CoV-2 antibodies, in particular rapid tests (RDTs) are a diagnostic complement to COVID-19. They have the advantage of being easy to realize, of being safe both in the laboratories and outside the laboratories. RDTs enabled us to detect asymptomatic SARS-CoV-2 carriers within CHN employees. This allowed for patients management and isolation to protect patients and their environments.

## Introduction

Fin décembre 2019, la première flambée d´une infection respiratoire d´étiologie inconnue a été signalée dans la ville chinoise de Wuhan. L´Organisation Mondiale de la Santé (OMS) a d’abord utilisé le terme « nouveau coronavirus 2019 » cette épidémie est actuellement appelée Coronavirus2 du syndrome respiratoire aigu sévère (SARS-CoV-2), tel que proposé par le Coronavirus Study Group (CSG) du comité international, le 11 février 2020 [1]. La maladie causée par le SRAS-CoV-2 a été par la suite baptisée par l'OMS « maladie à Coronavirus 2019 » ou COVID-19 [2]. Le SARS-CoV-2 appartient à un groupe de β-coronavirus, qui est un virus à ARN simple brin positif enveloppé du sous-genre *Sarbecovirus* (sous-famille: *Orthocoronavirinae)* [3].

Le terme coronavirus fait référence à la sous-famille des *Coronavirinae*, appartenant à la famille des *Coronaviridae*, elle-même faisant partie de l´ordre des *Nidovirales*. Les coronavirus infectent de nombreuses espèces mammifères et aviaires. Selon la taxonomie actuelle, les *Coronavirinae* sont subdivisés en quatre genres nommés *Alpha-, Beta-, Gamma-* et *Deltacoronavirus* [4]. Auparavant, six coronavirus ont été reconnus comme agent causal de l'infection chez l´homme. Parmi eux, les HCoV-229E et HCoV-NL63 appartiennent au genre *Alphacoronavirus*. Les quatre autres coronavirus appartiennent au genre *Betacoronavirus*, les HCoV-HKU1 et HCoV-OC43, le SARS-CoV et le MERS-CoV [5]. Il a été constaté que 96,2% de la séquence génomique du SARS-CoV-2 est identique à la BatCoV RaTG13. Le virus partage également 79,5% des séquences du génome avec SARS-CoV. Sur la base des preuves disponibles de séquençage du génome et de l’analyse évolutive, la chauve-souris a été suggérée comme réservoir naturel et origine du SARS-CoV-2. Les chauves-souris peuvent transmettre le virus à l'homme via des hôtes intermédiaires inconnus [1]. Le pangolin a également été suggéré de jouer un rôle dans l'infection virale en tant qu'hôte intermédiaire car il existe environ 99% de similitude de séquence entre les espèces de pangolins et SARS-CoV-2 [6].

Afin de lutter contre la pandémie de SRAS-CoV-2, il existe un besoin croissant et une demande d'outils de diagnostic complémentaires et différents de la réaction de polymérisation en chaîne de transcriptase inverse (RT-PCR) en raison de son coût et des difficultés de sa réalisation [7]. Un autre problème supplémentaire était le faible taux de positivité d'échantillons naso-pharyngés chez les patients présentant un syndrome clinique compatible avec COVID-19 dans la deuxième et la troisième semaine d'infection, qui est généralement la période au cours de laquelle les patients s'aggravent et sont admis à l'hôpital [8]. Récemment, des études publiées confirment l'utilité de combiner la PCR dans les exsudats naso-pharyngés et la détection des anticorps IgM et IgG dans le sang des patients. La combinaison des techniques à la fois moléculaire et sérologique ont permis à certains auteurs d'atteindre une sensibilité de 97% pour le diagnostic de l'infection par le SRAS -CoV-2 [8]. Le but de cette étude est le dépistage de SARS-CoV-2 par TDR d´une population des personnels de santé.

## Méthodes

Il s´agit d´une étude de dépistage organisée par le Centre Hospitalier National de Nouakchott pour son personnel. Elle a inclus 853 employés tous grades confondus (médecins, pharmaciens, infirmiers, secrétaires, personnels de sécurité, administrateurs…) dont 504 de sexe masculin et 331 de sexe féminin soit un sex-ratio de 1,52 avec un âge moyen de 39 ans et des extrêmes d´âge de 20 ans et 60 ans.

Le dépistage s´est déroulé pendant la semaine du 20/05/2020 au 27/05/2020. Ce dépistage à la recherche des anticorps IgG et IgM anti-SARS-CoV-2 a été réalisé par la technique immuno-chromatographique Biotime (Xiamen Biotime Biotechnology Co., Ltd.) ayant les caractéristiques suivantes: sur sang total, sensibilité: IgM 64,3%, IgG 96,4%, IgM + IgG 96,4%, spécificité IgM 100% et IgG 98,7%, IgM + IgG 98,7%%; sur le plasma, sensibilité: IgM 67,9%, IgG 96,4%, IgM + IgG 96,4%, spécificité IgM 96,7%, IgG 93,5%, IgM + IgG 93,5% et sur le sérum: la sensibilité IgM 81,7%, IgG 95,8% IgM + IgG 96,3%, spécificité IgM 98,3%, IgG 95%, IgM + IgG 95%.

Le test a été réalisé sur un prélèvement au bout du doigt après désinfection, en respectant les instructions du fabricant. Les tests positifs ont été répétés deux fois et aucune discordance n´a été constatée, tous les patients positifs ont bénéficié d´une RT-PCR sur un prélèvement naso-pharyngé.

## Résultats

Un total de 835 personnes ont été inclues dans notre étude, 14 personnes étaient positives, soit 1.67% des dépistés, dont 12 avaient des anticorps anti-SARS-CoV-2 de type IgM et IgG et deux (2) n´avaient que des IgM isolées. La PCR sur écouvillonnage naso-pharyngé a été réalisée chez ces 14 patients et s´est révélée positive chez 6 d´entre eux dont un n´avait que les IgM et 5 avaient des IgG et IgM. Les Tableau 1 et Tableau 2 récapitulent les principaux résultats de notre étude.

**Tableau 1:** données récapitulatives des principaux résultats

	Nombres	Pourcentage
Sexe		
Masculin	504	60.3%
Féminin	331	39,7%
Age		
Age moyen	39 ans	
Sérologie positive	14	1,67%

**Tableau 2:** résultats de la PCR chez les patients séropositifs

	RT-PCR positive	RT-PCR négative
Sérologie positive (14)	6	8
IgM + IgG (12)	5	7
IgM seules (2)	1	1

## Discussion

La RT-PCR pour la détection du SRAS-CoV-2 est considérée comme le gold standard pour le diagnostic de COVID-19. La RT-PCR a été utilisée pour analyser des échantillons des voies respiratoires supérieures et inférieures mais elle n'a pas pu être vulgarisée en milieu hospitalier car elle nécessite un équipement onéreux, un protocole qui prend du temps, des techniciens de laboratoire très qualifiés et des mesures de protection lors du prélèvement et au cours de la réalisation de cet examen. Par conséquent, la mise au point d´un test de diagnostic alternatif à la RT-PCR était nécessaire pour le diagnostic des patients COVID-19 [9]. Les tests sérologiques, s’ils sont faits au bon moment après le début de la maladie, peuvent détecter à la fois les infections actives et les infections guéries en fournissant des estimations sur l´immunité contre le SRAS-CoV-2 [10]. Ces tests présentent également l´avantage de pouvoir dépister un grand nombre d´individus dans un délai court d´où leur intérêt dans ce dépistage de masse que nous avons mené dans le milieu professionnel au Centre Hospitalier National de Nouakchott (CHN). Ce dépistage a révélé une prévalence de séropositivité de 1,67% (14/853) chez le personnel de santé du Centre Hospitalier National. Parmi ces 14 patients, 6 avaient une PCR sur prélèvement naso-pharyngé positive.

Dans notre étude, aucun patient n´avait des IgG isolées. Ce même résultat a été rapporté par Luca Bernasconi *et al*. [11]. L´absence des IgG isolées dans notre étude pourrait être expliquée par la courte durée entre le premier cas communautaire signalé à Nouakchott, Mauritanie, le 12/05/2020 et le déroulement de notre étude du 20/05/2020 au 27/05/2020. Les IgM augmentent après une semaine environ puis commence à décliner 4 à 5 semaines après le début de la maladie. Les IgG apparaissent juste après les IgM et semblent persister longtemps après une infection par le SRAS-CoV-2, cependant pour certains auteurs les IgG peuvent apparaitre avant les IgM [12]. Compte tenu de la spécificité du test pour la détection des IgM (100%) selon les données fournies par le fabriquant, tous nos patients séropositifs avaient contracté le SARS-CoV-2. Demey *et al*. n´ont retrouvé aucune réaction croisée avec les coronavirus humains pour cette technique immuno-chromatographique Biotime [13].

## Conclusion

Les tests sérologiques à la recherche des anticorps anti SARS-CoV-2 particulièrement les tests de diagnostic rapide (TDR) constituent un complément diagnostic du COVID-19, ils ont l´avantage de la facilité de réalisation, de leur sécurité aussi bien dans un laboratoire ou en dehors des laboratoires, dans les services ou en ville. Ces tests nous ont permis de de détecter des professionnels de santé porteurs asymptomatiques du SARS-CoV-2 dans notre institution, ce qui a permis de les isoler pour protéger les patients ainsi que leurs entourages.

### Etat des connaissances sur le sujet

- La RT-PCR reste la technique de référence pour le diagnostic de COVID-19;
- Les tests sérologiques ne deviennent positifs qu´environ une semaine après le début des symptômes.

### Contribution de notre étude à la connaissance

- Il s´agit de la première étude mauritanienne dépistant un groupe de professionnel hospitalier à la recherche des anticorps anti-SARS-CoV-2.

## Remerciements

Les auteurs tiennent à remercier l´équipe de l´INRSP: Pr Bollahi Med Abdallahi, Dr Ahmed Bara et leurs collaborateurs ainsi que Dr Mohamed Abdelkader.
